# Association between university aerobics course enrollment and changes in health-related physical fitness among college students: a retrospective cohort study

**DOI:** 10.3389/fpubh.2026.1957753

**Published:** 2026-09-17

**Authors:** Jun Liang, Yuanyuan Ma

**Affiliations:** 1 Nanchang Institute of Technology, Jiangxi, China; 2 Henan Finance University, Henan, China

**Keywords:** campus health promotion, course attendance, health-related physical fitness, physical education, retrospective cohort study, university aerobics

## Abstract

**Background:**

University aerobics is widely offered, but evidence comparing its real-world fitness outcomes with standard physical education (PE) using institutional records is limited. We examined whether enrollment in university aerobics was associated with greater 12-week improvement in health-related physical fitness than enrollment in standard PE and whether attendance was associated with fitness change.

**Methods:**

This retrospective cohort study used PE, attendance, and fitness-testing records for 240 undergraduates (120 aerobics, 120 standard PE), randomly sampled at 30 students per class from eight existing class sections (four aerobics and four standard PE). Week-0 and week-12 post-session measurements of vital capacity, 50-m sprint, sit-and-reach, standing long jump, sex-specific endurance run, and BMI were used to construct a sex-standardized composite fitness *z*-score. Between-group differences were estimated using ANCOVA adjusted for baseline outcome value, sex, age, grade year, and baseline BMI (except for BMI), with class-cluster-robust standard errors; wild-cluster bootstrap and random-intercept models were used as sensitivity analyses. Complete-case analyses included 232 students (96.7%).

**Results:**

The adjusted follow-up composite fitness score was 0.274 points higher in the aerobics group than in the standard PE group (cluster-robust 95% CI [0.225, 0.323]; *d* = 0.40). Cardiorespiratory endurance showed the largest secondary effect (*d* = 0.38), whereas standing long jump (*d* = 0.19), sit-and-reach (*d* = 0.17), 50-m sprint (*d* = −0.15), vital capacity (*d* = 0.10), and BMI (*d* = −0.08) showed small effects. Within the aerobics group, greater attendance was associated with greater composite fitness improvement (*β* = 0.050 points/session, 95% CI [0.028, 0.073]), although residual confounding cannot be excluded. No significant interactions by sex or baseline fitness were identified.

**Conclusion:**

Enrollment in the studied aerobics sections was associated with greater fitness improvement than enrollment in the available standard PE sections. Because course enrollment was non-randomized, only eight sections were available, the comparison condition was incompletely characterized, and relevant pre-exposure confounders were unavailable, the findings should be considered hypothesis-generating rather than causal.

## Introduction

Physical fitness among Chinese college students has continued to decline even as living standards have risen. Nationwide surveillance spanning five successive surveys from 2000 to 2019 shows a consistent decline in a composite physical fitness index alongside rising overweight/obesity and elevated blood pressure in this population, with the fitness decline itself tracking increases in cardiovascular risk (1); archival testing records from individual universities show the same downward pattern among more recent first-year cohorts (2). This trend has made improving campus-based physical activity a stated public health priority, and it places universities—which already run mandatory physical education (PE) coursework and routine fitness testing for nearly every undergraduate—in an unusually direct position to intervene on it.

That structural position has a specific theoretical basis. The social-ecological model of health behavior holds that individual behavior is shaped jointly by intrapersonal, interpersonal, and institutional or environmental factors, with institutions acting mainly by shaping opportunity and behavior rather than by producing health outcomes directly (3). Consistent with this framework, in social-ecological models of Chinese university students’ fitness specifically, physical activity itself, rather than institutional or family support in isolation, is consistently the strongest proximal correlate of measured fitness, with institutional factors such as PE curricula operating mainly indirectly, by shaping whether students actually engage in activity, rather than by improving fitness on their own (4). Required PE is therefore best understood as an institutional-level lever that works to the extent it changes students’ actual activity behavior—not merely their access to activity—and authors working with the same national fitness-testing infrastructure used in the present study have explicitly argued that PE courses built around moderate-to-vigorous-intensity activity are a natural avenue for addressing the declining-fitness trend (2).

Aerobics — structured, music-paced group exercise combining continuous aerobic movement with coordination and flexibility components—is one of the most widely offered PE formats built around sustained, moderate-to-vigorous activity of exactly this kind. Using the same national student physical fitness test battery applied in the present study (vital capacity, sprint, sit-and-reach, standing long jump, and endurance run; 5), a 16-week randomized trial of sports-dance training—a structurally similar, music-paced format—recently produced significant gains in core strength, lower-body power, agility, and flexibility among Chinese university students (5), and a 12-week program combining aerobic exercise with blood-flow-restriction training produced significantly greater gains than a no-additional-activity control in the endurance run, 50-m sprint, and standing long jump among Chinese high school students on this same test battery, though the aerobic-exercise-only comparison arm in that trial differed significantly from control only on standing long jump (6). Taken together, this evidence points toward endurance- and power-related indicators, rather than vital capacity or body composition, as the fitness components most likely to show an aerobics-specific advantage, while underscoring that direct, curriculum-level evidence in a Chinese university PE setting remains limited.

Nearly all of this evidence, however, comes from short, protocol-driven interventions delivered outside routine teaching — typically with dedicated recruitment, randomization, and purpose-built data collection — conditions that do not resemble how aerobics is actually taught as one of several parallel PE options within an ordinary academic term. Comparatively little evidence describes how aerobics performs as a routine curricular offering, evaluated against whatever other PE courses a university would otherwise deliver at the same scale, using the fitness-testing and attendance data institutions already collect for other administrative purposes. Retrospective designs built on exactly this kind of archival institutional data have recently been used to characterize fitness trends in Chinese university settings (1, 2), demonstrating that routinely collected records can support rigorous secular and comparative analysis without a dedicated research protocol; the present study extends this approach from descriptive trend-tracking to a controlled comparison between two concurrently taught course types.

Accordingly, this study asks whether students who completed a university aerobics course showed greater improvement in health-related physical fitness over one academic term than students who completed standard PE courses, drawing exclusively on institutional enrollment, attendance, and fitness-testing archives. As a secondary aim, we examined whether, among aerobics students, course attendance was associated with change in fitness; a similar attendance-linked association has previously been examined for a different aerobic training format using an analogous retrospective, archival design in a Chinese university setting (7). We hypothesized that (1) the aerobics group would show greater baseline-adjusted improvement in a composite health-related fitness score than the control group, with the largest expected advantage in endurance- and power-related outcomes and the smallest in BMI, broadly consistent with the pattern noted above (5, 6); and (2) among aerobics students, a greater number of sessions attended would be associated with greater fitness improvement.

## Materials and methods

### Study design

This was a retrospective controlled cohort study using institutional physical education course records and routine student fitness-test archives. Students had already been enrolled in either an aerobics course or a standard PE course by their institution before this study began; the investigator did not assign students to course type and had no role in course enrollment. The study compares the change in fitness from a week-0 post-session assessment (used as the analytic baseline) to a week-12 post-session assessment between these two pre-existing course groups, with statistical adjustment for measured baseline differences. Because both assessments were conducted after the scheduled class session, the observed change represents the change between two post-session assessments rather than a conventional pre-exposure to post-exposure comparison. Because course-group membership was not researcher-assigned, and because random selection of participants within class sections (Section 2.3) is not equivalent to random assignment to course type, results can only be interpreted as an association between course enrollment and fitness change, not as evidence of a causal course effect; causal language is avoided accordingly throughout this manuscript.

### Setting and data sources

Data were drawn from routine university PE enrollment records, course attendance logs, and the institution’s standardized student physical fitness testing program, which follows the national student physical fitness test battery used across Chinese universities (vital capacity, 50-m sprint, sit-and-reach, standing long jump, and a sex-specific endurance run; 4). The analytic records were obtained from the spring and autumn semesters of 2025; both the aerobics course and the standard PE sections were offered concurrently within each of the two semesters (i.e., both course types ran in the spring semester and both ran again in the autumn semester, rather than one course type being offered only in one semester), taught by the university’s regular PE faculty under the same institutional testing conditions in both semesters. Course-group assignment is therefore not confounded with semester at the design level, even though participant-level semester identifiers were not retained in the analytic dataset, and semester itself could not be entered as a covariate (Section 4.5). Each academic semester lasted 16 weeks, and routine fitness monitoring and recording were conducted after scheduled class sessions at weeks 0, 4, 8, and 12 of each semester as part of standard institutional practice, independent of this study. However, the analytic dataset made available for the present study retained only the week-0 and week-12 measurements; the week-4 and week-8 records were therefore not part of the analytic extract and could not be analyzed (Section 4.5). The present analysis accordingly covers a 12-week observation window, from a week-0 baseline assessment to a week-12 follow-up assessment, comprising 12 once-weekly, 45-min scheduled sessions; participant-level semester identifiers were not retained in the analytic dataset (Section 4.5). Because assessments at both timepoints were conducted after the scheduled class session rather than before it, acute exercise fatigue or short-term post-class recovery effects may have influenced performance at week 0 as well as at week 12; the available records did not specify a standardized post-exercise recovery interval before testing (Section 4.5). All students completing full-time undergraduate PE coursework are required to complete this fitness battery as part of standard institutional practice. Records were extracted for the period under review, de-identified, and matched across enrollment, attendance, and fitness-testing systems using an internal coded identifier before analysis; no names, student ID numbers, or other direct identifiers were available to the analytic team.

### Participants

Participants were drawn from eight existing class sections (four aerobics, four standard PE) at the institution. Within each of the eight class rosters, 30 students were randomly selected for inclusion in the analytic extract, yielding 120 students in the aerobics group and 120 in the standard PE group (240 total); the equal class and group sizes are a direct result of this class-stratified random sampling procedure and should not be interpreted as individual matching or as random assignment to course type — students were not randomly assigned to aerobics versus standard PE. The study size was determined by the available class-stratified analytic extract (30 randomly selected students per class section, eight sections); no *a priori* sample-size or power calculation was performed to determine this number. The specific random-number generation procedure used to select 30 students from each roster (e.g., software function, seed, or a random-number table) was not retained in the institutional records available for this analysis and is not asserted here; only the fact and stratification level of random selection are reported (Section 4.5). Eligible students within each class were full-time undergraduates with a baseline fitness record at course entry; a documented medical exemption from PE, duplicate or unmatched course/identifier records, or absence of a baseline fitness test would have excluded a student from the sampling frame, but exclusion counts by criterion and the size of the larger pre-exclusion class rosters were not available in the institutional records provided for this analysis (Section 4.5). Of the 240 sampled students, 232 (96.7%) had a valid week-12 follow-up assessment and were retained in the primary complete-case analysis: 117 aerobics (97.5%) and 115 standard PE (95.8%) (Figure 1). The eight without follow-up data were missing due to medical leave, course transfer, or absence from the final test (Section 3.7); missingness did not differ by group (Fisher’s exact test, *p* = 0.72).

**Figure 1:**
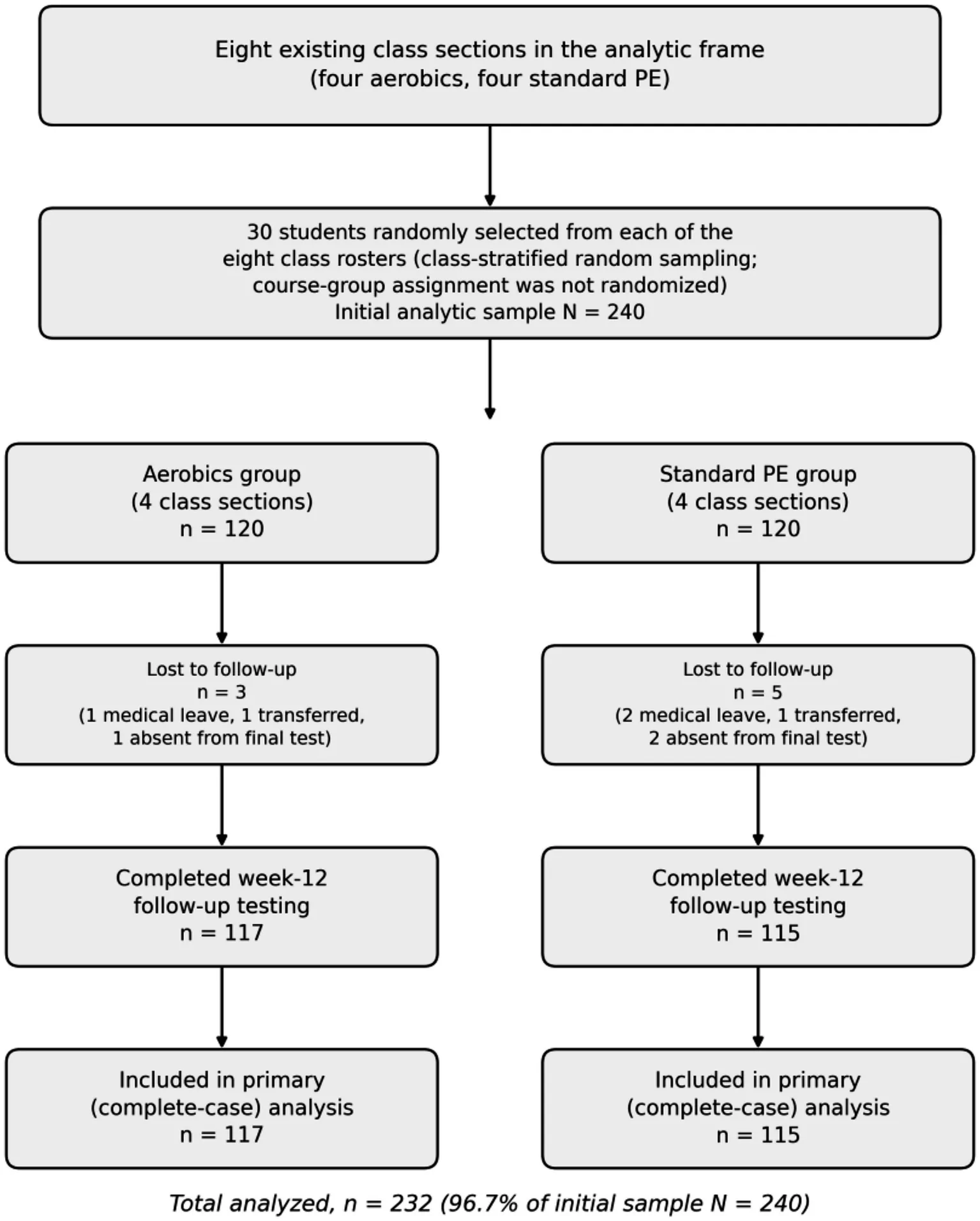
Participant flow through class-stratified random sampling, course participation, and analysis. Random selection occurred within class sections; course-group assignment was not randomized.

### Course descriptions

The aerobics course comprised 12 once-weekly sessions over the 12-week observation period (week 0 to week 12), with each session lasting 45 min, combining continuous aerobic movement with coordination, strength, and flexibility components, delivered across four class sections. The comparison group comprised four existing standard PE sections, with the same schedule of 12 once-weekly, 45-min sessions over the same observation period. The available analytic records identified course and section membership and attendance, but did not retain the specific sport or activity taught in each standard PE section, session intensity, instructor identity, or other section-level organizational characteristics. The comparison condition should therefore be interpreted as the available standard PE sections at this institution rather than as a content- or intensity-matched control, and observed between-group differences cannot be attributed specifically to the aerobics format. Class section was not entered as a conventional individual-level covariate in the statistical models, because course group was itself defined by class section (each section was entirely aerobics or entirely standard PE, so the two are collinear); instead, within-class dependence was addressed using class-cluster-robust standard errors and class-level sensitivity analyses (Section 2.6). Attendance in both course types was recorded by instructors using the university’s standard sign-in procedure.

### Variables

#### Exposure

Course group (aerobics = 1, standard PE = 0), coded from enrollment records at course entry and fixed for the term.

#### Anthropometry and BMI

Height (cm) and weight (kg) were recorded at baseline and follow-up as part of the standardized fitness-testing protocol. BMI was calculated as weight in kilograms divided by height in meters squared (kg/m^2^) at each time point, with height fixed at its baseline value. Equipment and calibration procedures are institutional testing-center details not contained in the data extract available for this analysis (Section 4.5).

#### Fitness indicators (instrument, unit, procedure)

All five indicators follow the national student physical fitness test battery (8): (1) vital capacity (mL), spirometer, best of trials per institutional protocol; (2) 50-m sprint (s); (3) sit-and-reach (cm), seated flexibility box test; (4) standing long jump (cm), best of trials; (5) endurance run—800 m women/1,000 m men (s), per national testing convention. Number of trials, timing method, and tester training/certification are testing-center procedural details not contained in the data extract available for this analysis (Section 4.5), consistent with STROBE guidance on measurement reporting (9).

#### Primary outcome: composite fitness *z*-score

To reduce the multiple-testing burden across the five correlated fitness indicators, a single composite score was constructed as follows. (1) For each indicator, sex-specific baseline reference means and standard deviations were computed from the full baseline sample (*n* = 240; baseline fitness values had no missing data), separately for men and women. (2) Each student’s baseline and follow-up value on each indicator was converted to a *z*-score using that indicator’s own sex-specific baseline mean and SD as the reference—i.e., follow-up values were standardized against the baseline reference distribution, not a separate follow-up distribution, so the resulting scores are directly comparable across time and reflect change relative to baseline. (3) The 50-m sprint and endurance-run *z*-scores were reverse-signed (multiplied by −1) so that, after this step, a higher *z*-score consistently indicates better fitness on every indicator. (4) The composite score at each time point is the unweighted (equal-weight) arithmetic mean of the five signed *z*-scores. Composite change is follow-up minus baseline composite score. Because the five fitness indicators had no missing values at baseline, and the eight students missing follow-up data were missing all five follow-up indicators simultaneously (Section 3.7), no case required a composite score to be computed from a partial subset of the five indicators; a per-case minimum-indicator or imputation rule for partial missingness was therefore not needed and was not used. The study was not preregistered. The composite fitness *z*-score and its designation as the primary outcome were finalized as part of the retrospective analysis rather than prospectively preregistered before access to the study data. Accordingly, the primary/secondary outcome distinction and the associated multiple-comparison framework should be interpreted as exploratory.

#### Secondary outcomes

BMI (kg/m^2^) and each of the five fitness indicators in their original units were analyzed separately in addition to the composite fitness score.

#### Covariates

Sex (male = 1, female = 0), age in years, grade year (ordinal, year of study), baseline value of the outcome under analysis, and baseline BMI, all drawn from institutional enrollment and fitness-testing records. Grade year was added as a model covariate (in addition to sex, age, and baseline BMI) because its baseline standardized mean difference between groups exceeded the threshold defined for the present analysis (0.20; Table 1); for the BMI outcome specifically, baseline BMI is not entered a second time as a separate covariate, since it is identical to the outcome’s own baseline value and including it twice would make the model’s design matrix singular (see Section 2.6). Age and grade year were correlated (*r* = 0.89), with variance inflation factors of 5.1 and 5.1 respectively, indicating meaningful collinearity between these two covariates specifically; the group indicator itself was not collinear with either (VIF = 1.02), and a sensitivity analysis refitting the composite model with each of age and grade year dropped in turn left the group coefficient essentially unchanged (0.274 with both covariates, 0.274 with age only, 0.273 with grade year only), so this collinearity does not materially affect the reported between-group estimates.

**Table 1:** Baseline characteristics by course group.

Characteristic	Aerobic (*n* = 120)	Control (*n* = 120)	SMD
Age, years, mean (SD)	19.66 (1.16)	19.41 (1.07)	0.22
Male sex, n (%)	47 (39.2)	50 (41.7)	−0.05
Grade year, mean (SD)	2.19 (1.03)	1.98 (0.99)	0.21
Baseline BMI, kg/m^2^, mean (SD)	21.67 (2.62)	21.78 (2.30)	−0.05
Baseline Vital capacity, mL, mean (SD)	3605.7 (752.4)	3578.2 (672.3)	0.04
Baseline 50-m sprint, s, mean (SD)	8.6 (0.7)	8.6 (0.7)	0.08
Baseline Sit-and-reach, cm, mean (SD)	14.8 (6.4)	14.4 (6.0)	0.08
Baseline Standing long jump, cm, mean (SD)	194.0 (28.6)	197.1 (28.3)	−0.11
Baseline Endurance run — Women, 800 m, s, mean (SD), *n*	250.88 (19.46), *n* = 73	250.24 (21.12), *n* = 70	0.03
Baseline Endurance run — Men, 1,000 m, s, mean (SD), *n*	266.09 (20.55), *n* = 47	271.58 (18.87), *n* = 50	−0.28
Baseline composite fitness *z*-score, mean (SD)	0.01 (0.69)	−0.01 (0.62)	0.03

SMD, standardized mean difference (Aerobics vs. Control); an absolute SMD >0.20 was considered a potentially meaningful imbalance and treated as a candidate covariate. The endurance run is reported separately by sex/distance (800 m women, 1,000 m men) because the two use different distances and are not comparable on a shared raw-seconds scale (Section 2.5); the men’s baseline imbalance on this item (SMD = −0.28) exceeds the 0.20 threshold, but raw endurance seconds are not used as a model covariate for any outcome, so this imbalance does not enter the adjusted models directly — it is addressed instead through the sex-standardized construction of the endurance and composite outcomes themselves (Section 2.5) and through sex as a covariate in all adjusted models (Section 2.6). “Baseline” refers to the week-0 post-session assessment used as the analytic baseline.

#### Course attendance/participation

Number of sessions attended out of 12 sessions scheduled, and the corresponding attendance rate (sessions attended/12), from course attendance logs. Because attendance is generated during the course rather than observed before it, it is reported and analyzed separately from baseline characteristics (Table 2) and is not entered as a covariate in the primary between-group ANCOVA models; it is used only in the aerobics-group-only exploratory attendance–fitness association analysis (Section 2.6).

**Table 2:** Course participation (not a baseline characteristic; generated during the term).

Characteristic	Aerobic (*n* = 120)	Control (*n* = 120)	SMD
Sessions scheduled	12	12	—
Sessions attended, mean (SD)	10.50 (1.26)	10.21 (1.17)	0.24
Attendance rate, %, mean (SD)	87.5 (10.5)	85.1 (9.8)	0.24

Sessions scheduled were fixed at 12 for both course types.

### Statistical analysis

Baseline characteristics are summarized by group as mean (SD) or *n* (%), with standardized mean differences (SMD) used alongside descriptive comparison to characterize baseline balance, following the convention that an absolute SMD greater than 0.20 was considered a potentially meaningful imbalance warranting adjustment. Course attendance and participation measures are summarized separately (Table 2) rather than as baseline characteristics, since they are generated during, not before, course participation.

The primary analysis was an ANCOVA-type linear model regressing the follow-up value of each outcome on group, the outcome’s own baseline value, sex, age, and grade year, fit separately for the composite score and each secondary outcome in the complete-case sample (*n* = 232); baseline BMI was included as an additional covariate for every outcome except BMI itself, where it is redundant with the outcome’s own baseline value and was therefore omitted from that one model to avoid a duplicated, perfectly collinear regressor. The coefficient on group is reported as the baseline-adjusted mean difference with a 95% confidence interval, alongside an approximate standardized effect size (adjusted mean difference divided by the follow-up outcome’s pooled standard deviation), interpreted using conventional small/medium/large benchmarks of 0.2/0.5/0.8 (10). Because participant-level semester identifiers were not retained in the analytic dataset (Section 2.2), semester was not entered as a covariate in any model, group×semester interactions were not tested, and no claim is made that potential semester-specific differences have been statistically accounted for (Section 4.5).

Because course type was assigned at the level of eight class sections (four aerobics, four standard PE) rather than at the level of individual students, ordinary model-based standard errors from a student-level ANCOVA cannot be assumed to be valid: the true number of independent course-delivery units is eight, not 232. To address this, each ANCOVA model was fit twice — once with conventional model-based standard errors, and once with cluster-robust (sandwich) standard errors clustered on class section (Python 3.12; pandas 3.0; statsmodels 0.14, using statsmodels.api. OLS with cov_type = “cluster” and cov_kwds = {“groups”: class_section}), with two-sided *p*-values computed using a t-distribution with 7 degrees of freedom (number of clusters−1), the conventional small-cluster convention — and both are reported in Table 3. As a further cross-check, a multilevel linear model with a class-level random intercept was fit for the composite outcome using statsmodels’ MixedLM, and the class-level intraclass correlation coefficient (ICC) is reported (Table 4). Because ordinary cluster-robust standard errors are known to be anti-conservative when the number of clusters is this small, we additionally implemented an exact two-sided wild-cluster bootstrap in Python (custom implementation, not a packaged library), following the small-cluster guidance in Cameron and Miller (11). For each outcome, we fit the group-restricted (null) model, applied Rademacher weights (±1) to its residuals at the class-cluster level, and reconstructed a bootstrap outcome as y* = fitted values (null model) + weighted residuals; because there are eight class clusters, there are exactly 2^8^ = 256 possible sign-permutation combinations, all of which were enumerated exactly (not sampled), and for each one the full model (with cov_type = “cluster”) was refit to obtain a bootstrap *t*-statistic. The two-sided bootstrap *p*-value is the proportion of these 256 replications whose bootstrap t-statistic was at least as extreme in absolute value as the *t*-statistic from the original (unpermuted) cluster-robust model. This exact enumeration has a structural floor on the smallest *p*-value it can produce: the all-same-sign replication (weights of +1 for every cluster) exactly reproduces the original data, so its bootstrap *t*-statistic always equals the observed *t*-statistic; its mirror replication (weights of −1 for every cluster) always ties it in absolute value. These two replications, therefore, always meet the “at least as extreme” threshold by construction, regardless of outcome, so the smallest achievable two-sided bootstrap *p*-value with eight clusters is 2/256 ≈ 0.008, not 1/256. We verified this floor directly (including checking, for every outcome, that all 128 sign-permutation pairs produced identical bootstrap-t magnitudes to within floating-point precision) and confirmed that all seven outcomes in Table 3 reach exactly this floor (2 of 256 permutations, *p* = 0.008); we report the exact count for each outcome in the Table 3 note rather than only the *p*-value, so that this resolution limit is transparent. We treat agreement across all four approaches (model-based, cluster-robust with small-sample degrees of freedom, multilevel, and wild-cluster bootstrap) as evidence that the primary conclusion is not an artifact of any single variance specification, but we flag explicitly that with only eight clusters (four per arm), none of these approaches—including the bootstrap’s coarse *p*-value resolution — can fully substitute for a design with more class sections; this caveat is carried into the Discussion and should be weighed by readers alongside the point estimates.

**Table 3:** Baseline-adjusted between-group differences (Aerobics vs. Control), ANCOVA models.

Outcome	Adj. difference	Model 95% CI	Model p	Cluster 95% CI	Cluster p	d	*n*	Holm *p*
Composite fitness *z*-score	0.274	[0.237, 0.311]	<0.001	[0.225, 0.323]	<0.001	0.4	232	— (primary outcome)
BMI, kg/m^2^	−0.211	[−0.283, −0.139]	<0.001	[−0.265, −0.157]	<0.001	−0.08	232	<0.001
Vital capacity, mL	71.794	[46.120, 97.468]	<0.001	[42.298, 101.291]	0.002	0.1	232	0.004
50-m sprint, s	−0.11	[−0.135, −0.085]	<0.001	[−0.127, −0.093]	<0.001	−0.15	232	<0.001
Sit-and-reach, cm	1.066	[0.771, 1.360]	<0.001	[0.601, 1.531]	0.003	0.17	232	0.004
Standing long jump, cm	5.344	[4.041, 6.646]	<0.001	[4.518, 6.169]	<0.001	0.19	232	<0.001
Endurance run (sex-standardized *z*-score)	0.410	[0.309, 0.510]	<0.001	[0.270, 0.549]	0.001	0.38	232	0.002

Adjusted models regress the follow-up value of each outcome on group, the outcome’s own baseline value, sex, age, grade year, and (for all outcomes except BMI) baseline BMI; the endurance run uses a sex-standardized z-score outcome (Section 2.5) rather than raw seconds, since raw times are not comparable across the two test distances. *p*-values are the model-based and class-cluster-robust p-values, respectively, the latter computed with a t-distribution and 7 degrees of freedom (number of clusters − 1), the conventional small-cluster convention (Section 2.6). An exact two-sided wild-cluster bootstrap (256 sign-permutation replications, full enumeration; Section 2.6) corroborated significance for every outcome in this table: for all seven outcomes, exactly 2 of 256 permutations (the all-same-sign replication and its exact mirror, which always reproduce and tie the observed statistic by construction) reached the observed cluster-robust *t*-statistic in absolute value (bootstrap *p* = 0.008 throughout); this is the smallest nonzero *p*-value this exact 256-permutation procedure can produce (Section 2.6). Holm-adjusted *p*-values (rightmost column) apply the Holm step-down correction across the six secondary/BMI outcomes; the composite score is the designated primary outcome and is not part of that correction family. Positive differences favor the aerobics group for vital capacity, sit-and-reach, standing long jump, the composite score, and the (already reverse-signed) endurance z-score; negative differences favor the aerobics group for BMI and 50-m sprint time — note that Figure 2 re-signs BMI and the sprint so that positive always denotes a better outcome for the aerobics group (with the BMI caveat noted there and in Section 3.3).

**Table 4:** Variance-specification sensitivity for the composite outcome.

Model	Adjusted mean diff	95% CI	*p*
Single-level ANCOVA (model-based SE)	0.274	[0.237, 0.311]	<0.001
Single-level ANCOVA (class-cluster-robust SE, 8 clusters)	0.274	[0.225, 0.323]	<0.001
Multilevel model (class-level random intercept)	0.274	[0.222, 0.327]	<0.001
Wild-cluster bootstrap	0.274	—	0.008

The wild-cluster bootstrap yields a two-sided *p*-value from test inversion rather than a model-based confidence interval; for the composite outcome, 2 of 256 exact sign-permutation replications (the all-same-sign replication and its exact mirror) reached the observed cluster-robust *t*-statistic in absolute value (*p* = 2/256 ≈ 0.008), the smallest nonzero p-value this exact procedure can produce (see Section 2.6 for implementation details).

For the present retrospective analysis, the composite score was treated as the main summary outcome and was not included in the Holm correction applied to the six secondary/BMI outcomes. Because this outcome hierarchy was not prospectively preregistered, the primary/secondary distinction and the corresponding multiplicity procedure should be interpreted as exploratory rather than confirmatory. Both unadjusted and Holm-adjusted significance are reported for these six outcomes in Table 3 and noted in Section 3.3, and secondary outcomes are interpreted as exploratory regardless of significance after correction.

Within the aerobics group, the exploratory association between sessions attended and composite fitness change was modeled with linear regression adjusting for sex, age, and baseline composite score, treating sessions attended as continuous; attendance was additionally examined descriptively across three categories (<75%, 75–89%, ≥90% of scheduled sessions, thresholds defined for the present analysis), with the low-attendance stratum (*n* = 5) reported descriptively rather than relied upon for inference given its small size (Section 3.5). Exploratory subgroup analyses tested group×sex and group×baseline-fitness-level interactions on composite fitness change; baseline fitness level was modeled both as a median-split binary indicator and, as an additional robustness check, as a continuous interaction between group and baseline composite score, since dichotomizing a continuous variable can obscure or distort a true continuous moderation pattern.

Given the low proportion of missing follow-up data (3.3%) and no evidence of differential attrition by group (Fisher’s exact test, chosen over a chi-square test given small expected cell counts), complete-case analysis was used as the primary approach, consistent with guidance that complete-case analysis is reasonable when data are missing at a low rate and there is no strong evidence against a missing-at-random mechanism, though multiple imputation can still improve precision even under those conditions (12); baseline characteristics of students with and without follow-up data were compared descriptively as a missing-data diagnostic. All analyses were conducted in Python (pandas, statsmodels); two-sided *p* < 0.05 was considered statistically significant. This report follows applicable items of the STROBE guidance for observational cohort studies (9).

### Ethical considerations

This study used de-identified, routinely collected institutional records. Course participation and fitness testing did not involve any additional intervention or testing beyond standard PE course participation and fitness testing conducted as part of the university’s mandatory curriculum in 2025, independent of this study. In 2026, following ethics approval, the author additionally contacted participants to obtain retrospective written informed consent for the secondary research use and sharing of their de-identified records; this was the only research-specific contact with participants. Data extraction and matching used coded identifiers, and no directly identifying information (name, student ID, contact details) was available to the analytic team. The retrospective research use of these de-identified records was reviewed and approved by the Institutional Ethics Committee of Henan Finance University, China (Ethical Approval No. 2026–008; approved 4 February 2026), consistent with the ethical standards of the Declaration of Helsinki (see Ethics statement).

## Results

### Participant flow and baseline characteristics

Thirty students were randomly selected from each of the eight class rosters (four aerobics, four standard PE), yielding an initial analytic sample of 240 students (120 aerobics, 120 standard PE); the equal group sizes reflect this class-stratified random sampling procedure rather than individual matching or random assignment to course type (Section 2.3). Exclusion counts by criterion and the size of the larger pre-exclusion class rosters were not part of the available institutional records for this analysis and are noted as a limitation (Section 4.5) rather than estimated. Of these 240 students, 232 (96.7%) completed week-12 follow-up fitness testing and were included in the primary analysis: 117 in the aerobics group (97.5%) and 115 in the standard PE group (95.8%) (Figure 1). Table 1 summarizes baseline characteristics. The two groups were similar on most baseline measures (absolute SMD < 0.20 for sex, BMI, and all five baseline fitness indicators), with somewhat higher age (SMD = 0.22) and grade year (SMD = 0.21) in the aerobics group; both were retained as covariates in the adjusted models (Section 2.6) rather than assumed to be balanced by design, consistent with the non-randomized nature of course-group membership. Course attendance, which is generated during rather than before the course, is summarized separately in Table 2 and was likewise somewhat higher in the aerobics group (SMD = 0.24).

### Primary outcome: composite fitness score

In the complete-case sample, the composite fitness score improved in both groups from the week-0 post-session assessment to the week-12 post-session assessment, but improved substantially more in the aerobics group (mean change 0.37 [SD 0.16] vs. 0.10 [SD 0.12] points on the composite *z*-score scale in the control group; Table 5). After adjusting for baseline composite score, sex, age, grade year, and baseline BMI, the aerobics group showed a significantly greater follow-up composite score than the control group (adjusted mean difference = 0.274 points on the composite scale; 95% CI [0.237, 0.311] with conventional model-based standard errors, 95% CI [0.225, 0.323] with standard errors clustered on the eight class sections; *p* < 0.001 either way). Because the composite score’s own population SD is about 0.6–0.7 rather than 1 (Table 1), this raw difference is not itself a standardized effect size; expressed relative to the composite’s own SD it corresponds to an approximate Cohen’s d = 0.40, a small-to-moderate effect (Table 3; Figure 2). The similarity of the two confidence intervals is reassuring, though with only eight clusters, neither should be treated as fully precise (Section 2.6; Limitations).

**Table 5:** Baseline, follow-up, and unadjusted within-group change by course group (complete-case sample, *n* = 232).

Outcome	Aerobics baseline	Aerobics follow-up	Aerobics change	Control baseline	Control follow-up	Control change
Composite fitness *z*-score	0.00 (0.69)	0.37 (0.70)	0.37 (0.16)	−0.02 (0.63)	0.07 (0.63)	0.10 (0.12)
BMI, kg/m^2^	21.66 (2.64)	21.43 (2.69)	−0.23 (0.31)	21.85 (2.30)	21.83 (2.32)	−0.02 (0.24)
Vital capacity, mL	3625.27 (750.90)	3733.50 (741.68)	108.22 (105.48)	3582.86 (684.28)	3623.21 (682.25)	40.35 (92.09)
50-m sprint, s	8.62 (0.74)	8.47 (0.74)	−0.15 (0.10)	8.57 (0.73)	8.53 (0.72)	−0.04 (0.09)
Sit-and-reach, cm	14.60 (6.24)	16.12 (6.39)	1.52 (1.25)	14.37 (6.03)	14.81 (6.15)	0.44 (0.96)
Standing long jump, cm	194.63 (28.72)	201.46 (28.71)	6.83 (5.45)	197.06 (28.51)	198.56 (28.40)	1.50 (4.43)
Endurance run, s (pooled across distances — see Table 6)	—	—	—	—	—	—

The endurance run uses different distances by sex (800 m for women, 1,000 m for men) under the national testing convention; a single pooled mean across both distances has no clear physiological meaning and is not reported here. Table 6 reports the endurance run separately by sex/distance; the composite fitness score correctly incorporates endurance performance via sex-specific standardization (Section 2.5) regardless of this raw-time reporting choice.

**Figure 2:**
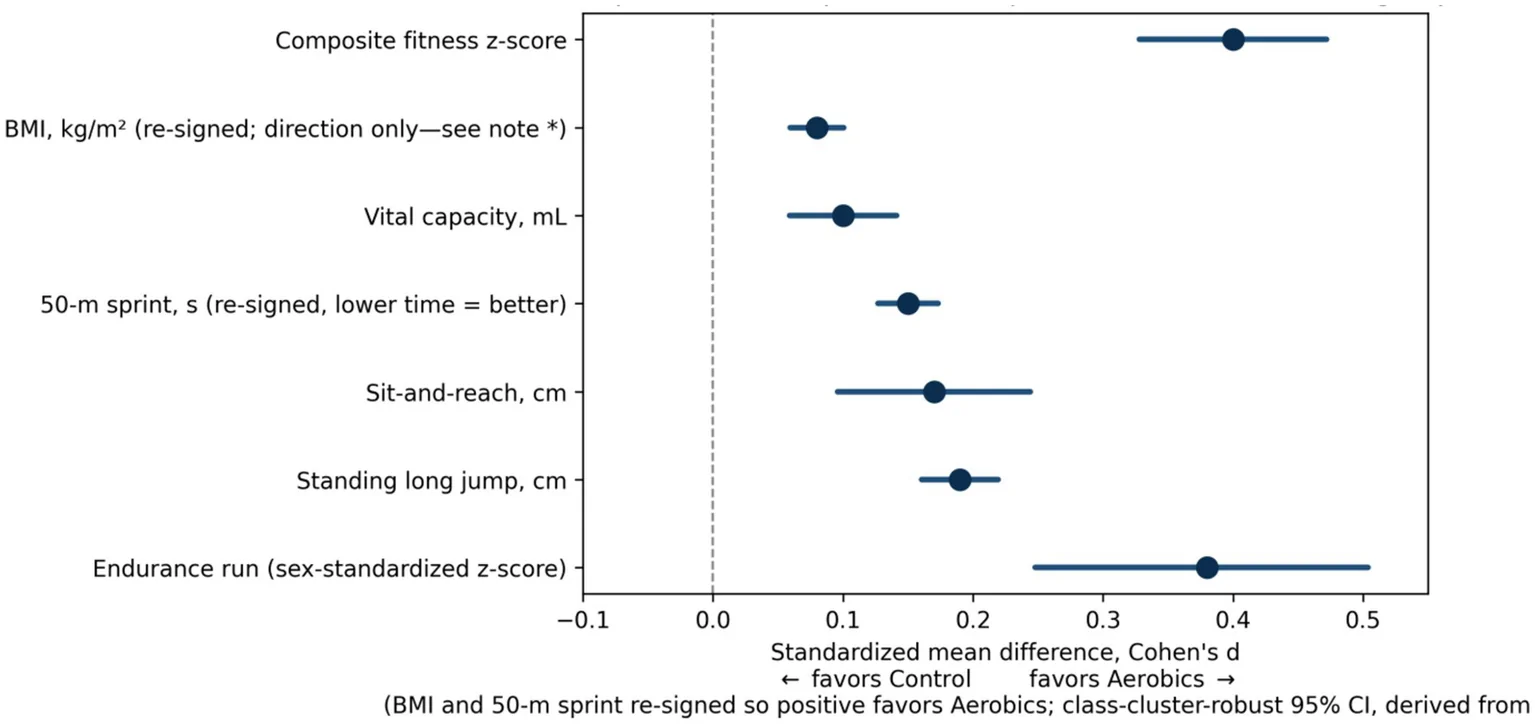
Baseline-adjusted between-group differences in fitness outcomes, expressed as standardized mean differences (Cohen’s *d*, derived from the adjusted mean differences and class-cluster-robust confidence intervals in Table 3) with class-cluster-robust 95% confidence intervals. BMI and the 50-m sprint have been re-signed so that, on this figure only, positive always indicates a better outcome for the Aerobics group; the endurance run is already reverse-signed via its sex-standardized *z*-score construction (Section 2.5). See the in-figure note for the caveat specific to the BMI estimate. ^*^BMI direction is re-signed for visual consistency only; because BMI is not monotonically “better when lower,” this point (*d* ≈ 0.08, a negligible effect) should be read as a directional change, not a health improvement (Section 3.3).

### Secondary outcomes

Table 5 reports baseline, follow-up, and unadjusted change values for each outcome by group, and Table 3 reports the corresponding baseline-adjusted between-group differences with both model-based and class-cluster-robust confidence intervals (the latter using a t-distribution with degrees of freedom equal to the number of clusters minus one, df = 7, the conventional small-cluster convention). The adjusted between-group differences varied in magnitude across outcomes. The largest secondary effect was observed for the endurance run (sex-standardized *z*-score, *d* = 0.38), whereas the effects for standing long jump (*d* = 0.19), sit-and-reach (*d* = 0.17), 50-m sprint (*d* = −0.15), vital capacity (*d* = 0.10), and BMI (*d* = −0.08) were small. All six secondary/BMI outcomes remained statistically significant after Holm correction for multiple comparisons, with the corresponding cluster-robust confidence intervals reported in Table 3. However, the small standardized effect sizes for most individual outcomes indicate that statistical significance should not be interpreted as evidence of large or practically important between-group differences. Because these are non-primary outcomes without a pre-registered ranking, they are best read as a consistent, exploratory pattern rather than as six independently confirmed findings. The very small effect size for BMI (d = −0.08), despite formal statistical significance, is unlikely to be practically meaningful over a single academic term and should not be over-interpreted; because BMI is not monotonically “better when lower” for students in a healthy weight range, this estimate is reported and plotted (Figure 2) as a directional change rather than as an unambiguous improvement. Similarly, the small effects observed for vital capacity, 50-m sprint, sit-and-reach, and standing long jump should be interpreted as modest between-group differences rather than as large improvements. The raw pooled-seconds endurance-run estimate is not reported in Table 3 because it mixes two different test distances (800 m women, 1,000 m men) within a single ANCOVA; instead, Table 3 reports a sex-standardized endurance *z*-score (constructed as in Section 2.5) as the unified secondary outcome. Fit separately by sex on the raw time scale, the adjusted between-group difference was −7.57 s among women (800 m; 95% CI [−10.63, −4.51], d = −0.34) and −9.18 s among men (1,000 m; 95% CI [−12.66, −5.70], *d* = −0.43); Table 6 reports the corresponding unadjusted descriptive values by sex/distance. Overall, the more substantial between-group differences were observed for the composite fitness score and cardiorespiratory endurance, whereas the remaining individual outcomes showed only small standardized effects.

**Table 6:** Endurance run, reported separately by sex/distance (complete-case sample).

Sex/distance	Aerobics baseline	Aerobics follow-up	Aerobics change	Control baseline	Control follow-up	Control change
Women, 800 m (Aerobic *n* = 70; Control *n* = 66)	251.6 (19.1)	241.3 (20.8)	−10.30 (8.23)	250.0 (21.5)	247.3 (23.1)	−2.77 (6.58)
Men, 1,000 m (Aerobic *n* = 47; Control *n* = 49)	266.1 (20.6)	255.6 (22.0)	−10.45 (7.91)	272.3 (18.3)	270.8 (18.5)	−1.49 (7.72)

### Cluster-level sensitivity analyses

Because course type was assigned at the level of eight class sections rather than 232 individual students, four complementary variance specifications were compared for the composite outcome (Table 4): the primary ANCOVA with conventional model-based standard errors, the same ANCOVA with standard errors clustered on class section (t-distribution, 7 degrees of freedom), a multilevel model with a class-level random intercept, and an exact wild-cluster bootstrap (256 sign-permutation replications). All four yielded the same point estimate (0.274), overlapping confidence intervals where a confidence interval applies, and a consistent conclusion (*p* ≤ 0.008 throughout), and the class-level intraclass correlation from the multilevel model was small (ICC ≈ 0.04), indicating that clustering by class section accounted for only a modest share of outcome variance. This agreement is reassuring, but it does not fully resolve the underlying issue: with only eight independent course-delivery units (four per arm), every one of these approaches is ultimately constrained by the limited number of clusters and the resulting uncertainty in cluster-level inference (11); this issue is discussed further in the Limitations section. Descriptive section-level results were consistent with the overall group pattern. Mean composite fitness change ranged from 0.302 to 0.418 across the four aerobics sections and from 0.085 to 0.113 across the four standard PE sections (Supplementary Table S1). Although this pattern indicates that the overall group difference was not attributable to a single section, section-level confounding cannot be excluded because course type did not vary within sections.

### Exploratory association between attendance and fitness change

Within the aerobics group, greater course attendance was associated with greater composite fitness improvement in an exploratory analysis. In an adjusted linear model, each additional session attended was associated with a 0.050-point increase in composite fitness score, on the composite *z*-score scale described in Section 2.5 (whose own population SD is about 0.6–0.7, not 1; this coefficient is therefore expressed in composite-score points, not composite-score SD units) (95% CI [0.028, 0.073], *p* < 0.001; Table 7); this continuous model is the primary basis for this exploratory analysis. As a purely descriptive comparison, mean composite fitness change was numerically higher in the medium-attendance group (75–89%, *n* = 48, mean = 0.32, SD = 0.17) and high-attendance group (≥90%, *n* = 64, mean = 0.41, SD = 0.14) than in the low-attendance group (<75%, *n* = 5, mean = 0.23, SD = 0.15; Figure 3). However, because the low-attendance group included only five students, this categorical comparison is not a reliable basis for inference. The associated one-way ANOVA (*F* = 7.32, *p* = 0.001; Table 7) is reported for completeness only and should not be interpreted as confirmatory. This association should not be interpreted as a causal per-session effect: students who attend more sessions may also differ systematically in ways not captured by our covariates — for example, being in better baseline health, more motivated toward exercise generally, or less prone to illness or competing time demands — any of which could produce an attendance–fitness association independent of a true per-session training effect. This pattern is conceptually analogous to the “healthy-adherer” effect documented in pharmacological research, where good adherence to treatment — and even to placebo — predicts better outcomes than poor adherence, indicating that adherence itself can be a marker of unmeasured healthy behavior rather than proof of a treatment mechanism (13); we cite this only as a conceptual analogy from the medication-adherence literature, not as direct evidence from a PE-course population. We therefore treat the entire attendance analysis, continuous and categorical alike, as exploratory evidence of an attendance–fitness association rather than evidence of a causal per-session effect (see Limitations).

**Table 7:** Exploratory association between aerobics course attendance and composite fitness change.

Analysis	Estimate	95% CI	*p*	*n*
Continuous: sessions attended (per session)	*β* = 0.050 composite-score points/session	[0.028, 0.073]	<0.001	117
Low attendance (<75%)	mean change 0.234 (SD 0.150)	—	—	5
Medium attendance (75–89%)	mean change 0.316 (SD 0.173)	—	—	48
High attendance (≥90%)	mean change 0.414 (SD 0.141)	—	—	64
One-way ANOVA across attendance categories (descriptive comparison only; low-attendance cell n = 5, not relied upon for inference)	*F* = 7.32	—	0.001	117

**Figure 3:**
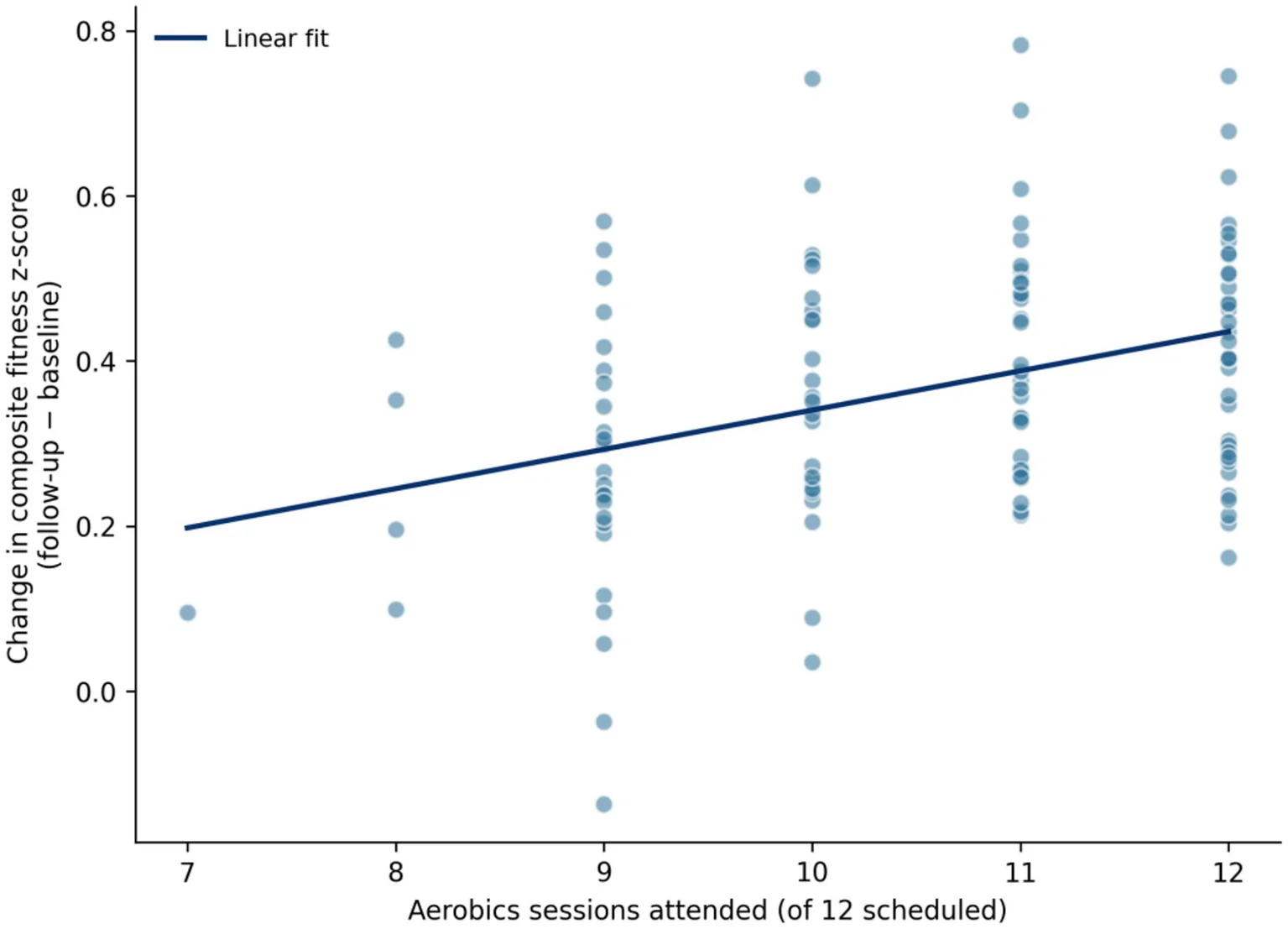
Association between aerobics course attendance and composite fitness change (aerobic group, *n* = 117).

### Subgroup analyses

Exploratory subgroup analyses found no evidence that the aerobics course effect on composite fitness change differed by sex (group×sex interaction coefficient = 0.013, 95% CI [−0.063, 0.088], *p* = 0.74; female: aerobics 0.376 [0.157] vs. control 0.111 [0.117]; male: aerobics 0.351 [0.173] vs. control 0.074 [0.121]; Table 8). Baseline fitness level was examined in two ways. A median split found a numerically larger aerobics-vs-control difference among students below the baseline median (0.391 [0.145] vs. 0.103 [0.110]) than at or above it (0.343 [0.177] vs. 0.088 [0.130]), but the group×baseline-level interaction was not significant (coefficient = −0.035, 95% CI [−0.109, 0.039], *p* = 0.35). As a robustness check, we additionally modeled baseline composite fitness as a continuous moderator rather than dichotomizing it; this continuous interaction was essentially null (coefficient = 0.001, 95% CI [−0.056, 0.058], *p* = 0.97), which does not support the median-split analysis’s suggestion that lower-baseline-fitness students benefited more, and we weight the continuous result more heavily since dichotomization can manufacture the appearance of a moderation pattern that a continuous model does not corroborate. Taken together, these null interaction tests do not provide evidence that the aerobics-versus-control advantage differs by sex or by baseline fitness level; a null interaction test is not proof of equivalent effects across subgroups, particularly given the modest power available for interaction terms in a sample of this size, and this absence of evidence should not be read as evidence of no difference.

**Table 8:** Subgroup analyses of composite fitness change.

Subgroup	Aerobic change, mean (SD), *n*	Control change, mean (SD), *n*
Female	0.376 (0.157), *n* = 70	0.111 (0.117), *n* = 66
Male	0.351 (0.173), *n* = 47	0.074 (0.121), *n* = 49
Baseline fitness below the median	0.391 (0.145), *n* = 56	0.103 (0.110), *n* = 60
Baseline fitness at/above median	0.343 (0.177), *n* = 61	0.088 (0.130), *n* = 55

Group×sex interaction *p* = 0.74. Group×baseline-fitness-level interaction: *p* = 0.35 (median-split) and *p* = 0.97 (continuous baseline composite score as moderator).

### Missing data

Eight of 240 students (3.3%) lacked follow-up fitness data (3 aerobics, 5 control), due to medical leave (*n* = 3), course transfer (*n* = 2), or absence from the final test (*n* = 3); for all eight, every one of the five follow-up fitness indicators was missing together (i.e., no case had a partially missing follow-up record), so no per-indicator imputation rule was required for the composite score (Section 2.5). Missingness did not differ significantly by course group (Fisher’s exact test, *p* = 0.72, preferred over a chi-square test given the small cell counts). Students missing follow-up data did not differ significantly from completers on baseline BMI (*p* = 0.38) or baseline composite fitness (*p* = 0.13), though they were somewhat older on average (20.4 vs. 19.5 years, *p* = 0.054). Given the low missing-data proportion and lack of strong evidence for differential attrition, complete-case analysis was judged adequate as the primary approach; multiple imputation was not performed.

## Discussion

### Principal findings

In this retrospective, real-world comparison of two routinely offered university PE course formats, students enrolled in an aerobics course showed significantly greater baseline-adjusted improvement in a composite health-related fitness score than students enrolled in standard PE courses over one academic term (*d* ≈ 0.40), with the largest single-indicator advantage in the endurance run and the smallest in the BMI-related outcome. The composite advantage held up under both cluster-robust and multilevel sensitivity analyses, accounting for class-section clustering, and within the aerobics group, fitness improvement was associated with the number of sessions attended — an exploratory attendance–fitness association that does not establish a causal per-session effect, since students who attend more sessions may also differ systematically in unmeasured ways (a healthy-adherer pattern; Section 3.5, Limitations). Subgroup analyses did not find evidence that this advantage differed by sex or by students’ starting fitness level, though the absence of a significant interaction is not proof that effects are truly equal across these subgroups.

### Comparison with existing literature

The direction and general magnitude of these findings are broadly consistent with previous aerobic-exercise research using the same national fitness-test battery in Chinese secondary-school samples, although important differences exist between the study designs. Jiang and Che (6) randomized high school students to aerobic exercise combined with blood flow restriction training, aerobic exercise alone, or no additional activity over 12 weeks. Compared with the no-additional-activity control, the combined intervention produced significantly greater improvements in the endurance run, 50-m sprint, and standing long jump. The aerobic-exercise-alone group, which more closely resembles the course format examined in the present study, differed significantly from the control group only in the standing long jump, although substantial within-group improvements were also observed. These findings suggest that aerobic training may benefit endurance- and power-related fitness indicators, but the use of blood flow restriction and a secondary-school sample limits direct comparison with routine university aerobics courses.

A retrospective dose–response analysis of a university running program found that greater training exposure was associated with greater improvement in aerobic fitness (7). Although that study used a single-arm pre-post design without a standard PE comparison group, its findings are consistent with the attendance–fitness association observed in the present study. The present study extends this literature by comparing aerobics with concurrently delivered standard PE courses using routinely collected institutional records.

More broadly, a network meta-analysis of exercise interventions in sedentary adults found that aerobic training ranked highest among exercise modalities for improving peak oxygen uptake (14). Although the population and outcome measures differed from those in the present study, this evidence provides a general physiological context for the comparatively larger difference observed in cardiorespiratory endurance. Direct evidence comparing routinely delivered university PE course formats nevertheless remains limited.

### Possible explanations

Several non-mutually-exclusive mechanisms could plausibly explain a greater fitness benefit from aerobics relative to standard PE courses, though this study did not measure them directly, and they should be read as hypotheses rather than demonstrated pathways. Aerobic sessions are structured around sustained, rhythmic, continuous movement, which may deliver a more consistent aerobic training stimulus per session than PE formats built around intermittent activity (e.g., skill practice, game play with variable exertion). The standardized, music-paced format may also support more consistent session-to-session intensity and completion, which is broadly consistent with the observed relationship between attendance and fitness gain. Group-based, choreographed exercise may additionally support adherence through social and motivational factors that are not captured in attendance counts alone. Because the endurance run showed the largest secondary-outcome effect, one plausible explanation among these candidates is an aerobic-conditioning pathway specific to cardiorespiratory endurance, though we cannot distinguish this from the alternatives listed above with the data available; the modest BMI-related effect suggests that a single term of coursework, however structured, is not sufficient to produce large changes in weight-for-height status.

### Public health and educational implications

University PE reaches a large proportion of the undergraduate population and therefore provides an important setting for evaluating campus-based approaches to physical fitness. In the present study, students enrolled in the studied aerobics sections showed greater adjusted improvement in health-related fitness than students enrolled in the available standard PE sections, with the more substantial differences observed for composite fitness and cardiorespiratory endurance. However, because course enrollment was not randomized, only eight course sections were available, and the specific content and intensity of the standard PE sections were not fully characterized, these findings do not provide a sufficient basis for recommending reallocation of curricular resources toward aerobics.

Rather, the observed between-group differences support further prospective evaluation of aerobics and other well-characterized PE formats. Future studies using prospectively defined comparison conditions, more course sections, and preferably randomized or otherwise stronger designs are needed before conclusions can be drawn regarding whether aerobics should be preferentially incorporated into university PE curricula.

## Limitations

This study has several limitations that bear on internal validity and on what the available institutional data can support. Course enrollment was not randomized: students self-selected or were administratively assigned into aerobics versus standard PE, and unmeasured differences between groups (e.g., baseline exercise motivation, prior athletic experience, out-of-class activity) could confound the observed association even after covariate adjustment (15); propensity-score methods were not feasible given the available pre-enrollment covariates (16). Random sampling of 30 students per class roster (Section 2.3) improved consistency within course groups but did not address this between-group selection bias. Second, the true unit of treatment assignment was the class section, and only eight sections (four per course type) were available at a single institution. Cluster-robust standard errors, a class-level random intercept, and an exact wild-cluster bootstrap (Sections 2.6, 3.4) all agreed closely, but variance estimates can remain imprecise with this few clusters (11); a design with more sections per arm, or a cluster-randomized extension, would allow greater confidence in the inferential statistics.

The exploratory attendance–fitness association (Section 3.5) does not establish a causal per-session effect, since higher-attending students may have differed systematically from lower-attending students in baseline health or motivation (a healthy-adherer pattern; 13); this should be read as hypothesis-supporting rather than hypothesis-confirming. Fourth, several details needed to fully judge external validity and course comparability were not part of the available data extract and are not asserted here: the standard PE curriculum, schedule, and intensity; instructor identity and qualifications; the university’s identity beyond its stated affiliation; and testing-center procedural details (Section 2.5). Fitness was assessed at weeks 0, 4, 8, and 12, but only the week-0 and week-12 measurements were retained, so the trajectory of change within the term could not be examined; although both course types were offered concurrently within each of the spring and autumn 2025 semesters (Section 2.2), which limits the risk of course type being systematically confounded with semester, participant-level semester identifiers were also not retained, preventing adjustment for any residual semester-specific factors.

Out-of-class physical activity, diet, sleep, and other lifestyle factors were not measured. Sixth, the endurance run differs in distance by sex (800 m women, 1,000 m men) by national convention; standardizing within sex addressed this but limits direct cross-sex comparison of raw times. Seventh, both baseline and follow-up assessments were conducted after the scheduled class session rather than before it, so acute exercise fatigue may have influenced performance at either time point, possibly non-differentially or differentially across groups; the direction and magnitude of any such effect could not be determined, and the week-0 assessment is therefore not a strictly unexposed baseline. Finally, missing follow-up data were limited (3.3%) and not clearly differential by group, though the low-attendance subgroup (n = 5) was too small to support a stable estimate. Because of these limitations, this study should be read as evidence of a real-world association consistent with a beneficial course effect, not as confirmation of a causal effect of aerobics instruction; a stepped-wedge, cluster-randomized, or propensity-matched multi-term extension — with more class sections and documented course content — would substantially strengthen both causal and reporting validity.

## Conclusion

Using routinely collected institutional records, this retrospective cohort study found that university students enrolled in aerobics sections showed greater improvement in health-related physical fitness, particularly cardiorespiratory endurance, than students enrolled in standard PE sections over one academic term. Within the aerobics group, greater attendance was associated with greater fitness improvement, although residual confounding, including a healthy-adherer explanation, cannot be excluded. Because the specific content and intensity of the standard PE comparison sections were not fully characterized, part of the observed difference may reflect variation in comparison-course content rather than the aerobics format itself.

These findings support further evaluation of aerobics as a potentially scalable campus-based approach to physical fitness promotion and demonstrate the potential use of routinely collected PE and fitness-testing records for ongoing evaluation of curricular health outcomes. Given the non-randomized, single-institution design, the small number of class sections, and the absence of relevant pre-exposure confounders, the findings should be regarded as hypothesis-generating. Prospective studies involving more institutions and academic terms, well-characterized comparison conditions, and stronger designs, such as propensity-matched or cluster-randomized studies, are needed before the findings can inform formal curricular policy.

## Data Availability

The original contributions presented in the study are included in the article/Supplementary material, further inquiries can be directed to the corresponding author.
