# Supplementary material for: Association between university aerobics course enrollment and changes in health-related physical fitness among college students: a retrospective cohort study
Source: Front Public Health. 2026 Sep 17;14:1957753. doi: 10.3389/fpubh.2026.1957753 (PMC13627356; doi:10.3389/fpubh.2026.1957753)
Supplement: Supplementary file 1 [file Table_1.docx]

Supplementary Table S1. Section-specific change in composite fitness score.

| Section | Group | Sampled n | Complete-case n | Composite fitness change, mean (SD) |
| --- | --- | --- | --- | --- |
| AER-01 | Aerobics | 30 | 29 | 0.418 (0.160) |
| AER-02 | Aerobics | 30 | 30 | 0.302 (0.163) |
| AER-03 | Aerobics | 30 | 28 | 0.370 (0.116) |
| AER-04 | Aerobics | 30 | 30 | 0.377 (0.190) |
| CON-01 | Standard PE | 30 | 30 | 0.113 (0.117) |
| CON-02 | Standard PE | 30 | 30 | 0.085 (0.107) |
| CON-03 | Standard PE | 30 | 28 | 0.090 (0.129) |
| CON-04 | Standard PE | 30 | 27 | 0.094 (0.129) |
